# Central Venous Pressure Revisited: Physiology, Pitfalls, Misconceptions, and Modern Clinical Interpretation in Critical Care

**DOI:** 10.3390/jcm15114227

**Published:** 2026-05-30

**Authors:** Cesare Biuzzi, Elena Modica, Lucrezia Pondrelli, Alexander Raimondi, Margherita Cavenago, Daniele Marianello, Filippo Annoni, Fabio Silvio Taccone, Federico Franchi, Sabino Scolletta

**Affiliations:** 1 Department of Medical Science, Surgery and Neurosciences, Urgency-Emergency Anesthesia and Intensive Care Unit, University Hospital of Siena, 53100 Siena, Italy; cesare.biuzzi@ao-siena.toscana.it (C.B.); dott.elena.modica@gmail.com (E.M.); lucrezia.pondrell@student.unisi.it (L.P.); alexander.raimondi@student.unisi.it (A.R.); margherita.cavenago@ao-siena.toscana.it (M.C.); 2 Department of Medical Science, Surgery and Neurosciences, Cardiothoracic and Vascular Anesthesia and Intensive Care Unit, University Hospital of Siena, 53100 Siena, Italy; daniele.marianello@unisi.it (D.M.); federico.franchi@unisi.it (F.F.); 3 Department of Intensive Care, Hôpital Universitaire de Bruxelles (HUB), Université Libre de Bruxelles (ULB), Route de Lennik, 808, 1070 Brussels, Belgium; filippo.annoni@hubruxelles.be (F.A.); fabio.taccone@ulb.be (F.S.T.)

**Keywords:** central venous pressure, fluid responsiveness, venous congestion, hemodynamic monitoring, critical care, sepsis resuscitation

## Abstract

Central venous pressure (CVP) has long been a cornerstone of hemodynamic monitoring, traditionally interpreted as a surrogate of intravascular volume and cardiac preload. Current evidence demonstrates that CVP has limited value as a standalone marker of preload and fluid responsiveness (FR), and its role as a fixed target for fluid resuscitation has progressively declined. This narrative review retraces the evolution of CVP interpretation, from its physiological foundations to its role in contemporary clinical practice. While early resuscitation strategies relied on predefined CVP thresholds, this approach has been abandoned. Despite these limitations, CVP remains widely used due to its simplicity and historical familiarity and modern perspectives instead emphasize its role as a marker of venous congestion. In this context, CVP retains clinical utility when used for waveform interpretation, assessment of venous congestion, and, most importantly, as part of an integrated, multimodal hemodynamic monitoring strategy.

## 1. Introduction

Central venous pressure (CVP) is traditionally regarded as one of the most commonly used parameters for evaluating volume status in critically ill patients, typically measured via a central venous catheter (CVC) placed in the internal jugular or subclavian vein, with its tip positioned in the lower superior vena cava or at the cavo-atrial junction. CVP represents the intraluminal pressure within the thoracic vena cava in close proximity to the right atrium and is commonly used as an estimate of the right atrial pressure (RAP) under appropriate physiological conditions [1]. Physiologically, CVP reflects the dynamic interaction between venous return and right-heart function and is influenced by several factors, including intrathoracic pressure, venous tone and compliance, right ventricular compliance, afterload and tricuspid valvular function. Importantly, as CVP is a pressure measurement and is influenced by these factors, it does not directly measure intravascular volume or ventricular preload [2].

Because CVP is readily available, continuously measurable, and easily obtained in most critically ill patients, it remains widely used in clinical practice [3]. In critically ill patients, CVP waveform analysis is mainly used in patients with shock, right ventricular dysfunction, pericardial disease, significant arrhythmias or tricuspid valve pathology, sepsis or multiorgan failure, after cardiac surgery or cardiac arrest, and in those with complex fluid management, such as renal failure or at high risk of fluid overload. In this setting, CVP remains a readily available and continuously measurable parameter that contributes to hemodynamic assessment when interpreted within a multimodal framework [4].

However, despite its apparent simplicity and longstanding integration into clinical algorithms, CVP is a highly controversial physiological variable in contemporary critical care. Over more than 70 years, CVP has progressed from an experimental parameter to a central component of fluid resuscitation strategies. Initially adopted as a stand-alone marker of intravascular volume and a fixed resuscitation endpoint, its reliability has been increasingly challenged [2]. As a result, the contemporary interpretation of CVP has shifted from a surrogate of preload to a marker of venous congestion and right ventricular loading conditions [1,2,5,6,7].

This narrative review was conducted in accordance with the SANRA recommendations for narrative reviews (Appendix A). A focused literature search was performed using PubMed, Embase, and the Cochrane Library for articles published up to February 2026. Search terms included combinations of ‘central venous pressure’, ‘CVP’, ‘fluid responsiveness’, ‘venous congestion’, ‘hemodynamic monitoring’, ‘right atrial pressure’, and ‘critical care’. We prioritized physiological studies, observational and interventional clinical studies, meta-analyses, and international guidelines published in English. Article selection was based on relevance to physiology, critical care hemodynamics, venous congestion, and contemporary clinical interpretation of CVP. Given the narrative nature of the review, no formal risk-of-bias assessment or systematic study selection process was performed. While recent narrative reviews have primarily focused on the physiological interpretation and technical limitations of CVP measurement, the present review specifically emphasizes the contemporary integration of CVP into multimodal hemodynamic assessment, including venous congestion evaluation, VExUS interpretation, waveform physiology, and investigational applications such as CVP-derived pleural pressure estimation. In this context, our aim was to examine recent literature to highlight how current routine clinical practice often diverges from contemporary physiological evidence and pathophysiological understanding.

## 2. CVP Measurement and Interpretation

**Figure 1:**
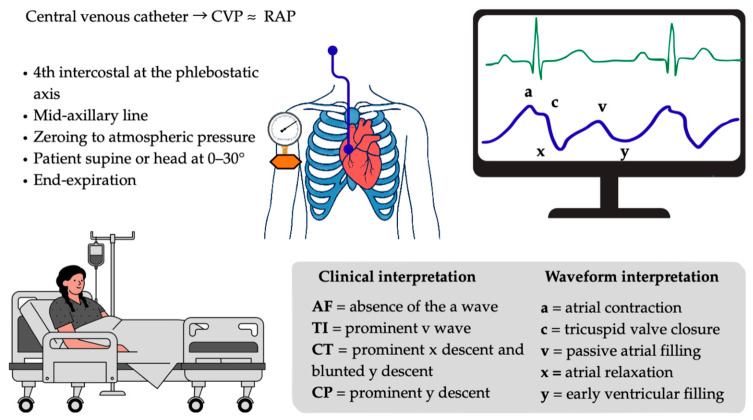
Central venous pressure (CVP) is measured via a central venous catheter with the pressure transducer positioned at the phlebostatic axis (fourth intercostal space at the mid-axillary line), corresponding to the level of the right atrium, and zeroed to atmospheric pressure. Measurements should be obtained with the patient in the supine position or with the head elevated at 0–30°, and recorded at end-expiration to minimize the influence of intrathoracic pressure variations. The CVP waveform reflects right atrial pressure (RAP) over time and consists of characteristic components: the a wave (atrial contraction), c wave (tricuspid valve closure and early ventricular systole), and v wave (atrial filling during ventricular systole). The x descent represents atrial relaxation and downward displacement of the tricuspid valve, while the y descent reflects early ventricular filling. Interpretation of the CVP waveform provides clinically relevant information beyond mean pressure values and should be integrated with respiratory conditions, cardiac rhythm, and overall hemodynamic context. Alterations in waveform morphology may provide diagnostic clues to specific pathophysiological conditions such as atrial fibrillation (AF), tricuspid regurgitation (TR), pericardial tamponade (PT), or constrictive physiology (CP).

Because CVP represents a pressure generated by the interaction of multiple cardiovascular and respiratory factors, its interpretation requires integration of numerical values, waveform morphology, respiratory conditions, and the overall hemodynamic context. Accurate CVP measurement requires positioning the transducer at the phlebostatic axis (fourth intercostal space at the mid-axillary line, approximating right atrial level), zeroing it to atmospheric pressure, and interpreting both the mean pressure and waveform morphology [1,8]. The CVP waveform reflects the time-varying pressure within the right atrium and central venous system and classically consists of the a, c, and v waves, together with the x and y descents [2]. The a wave corresponds to right atrial contraction and becomes more prominent when atrial emptying faces increased resistance. The c wave occurs in the early ventricular systole and reflects tricuspid valve closure and bulging of the valve into the right atrium. The v wave represents right atrial filling during ventricular systole and may become accentuated in tricuspid regurgitation. The x descent reflects atrial relaxation and systolic downward displacement of the tricuspid annulus; a preserved or prominent x descent suggests intact atrial relaxation and longitudinal right ventricular shortening, whereas a blunted x descent may be seen in severe tricuspid regurgitation and can accompany right ventricular systolic dysfunction. The y descent reflects early diastolic emptying of the right atrium into the right ventricle; it is typically prominent in constrictive physiology or severe tricuspid regurgitation and blunted in cardiac tamponade [2,9,10]. Measurements should be obtained with patient supine or with the head at 0–30°, without external compression on the catheter and at end-expiration, particularly in mechanically ventilated patients, as intrathoracic pressure swings can significantly influence the recorded value [1] (Figure 1).

The analysis of CVP waveforms provides valuable physiological insights beyond the absolute pressure value, allowing clinicians to infer underlying cardiac function and hemodynamic states. Alterations in specific components of the wave provide pathophysiological signatures of peculiar conditions, including loss of atrial systole (e.g., atrial fibrillation), atrioventricular dissociation (cannon a-waves), increased right atrial pressure due to systolic backflow (prominent v-waves in tricuspid regurgitation), impaired right ventricular filling (blunted y-descent), or enhanced early diastolic filling under constrictive physiology (steep y-descent). Similar waveform alterations may also be observed during ventricular ectopic beats, in which transient atrioventricular dissociation can produce intermittent cannon a-waves and irregular venous pulse contours [4,11] (Table 1).

A critical aspect often overlooked in clinical practice is that CVP does not represent a single piece of information, but rather a composite signal from which different types of physiological data can be derived [1,4,8]. Beyond waveform morphology, these include: (i) the absolute value, reflecting right atrial pressure at a given time point; (ii) temporal trends, providing insight into the dynamic response to interventions such as fluid administration; (iii) respiratory variations, which reflect heart–lung interactions and changes in intrathoracic pressure [12,13,14]. Each of these components conveys distinct physiological information and has different clinical implications. Accordingly, many limitations attributed to CVP arise from focusing exclusively on its absolute value, while neglecting its dynamic and morphological features. A comprehensive interpretation of CVP therefore requires integration within the overall hemodynamic context, including cardiovascular and respiratory conditions and potential artifacts (Table 2) [3,10,15].

## 3. Historical Perspective and Physiological Basis of CVP

**Figure 2:**
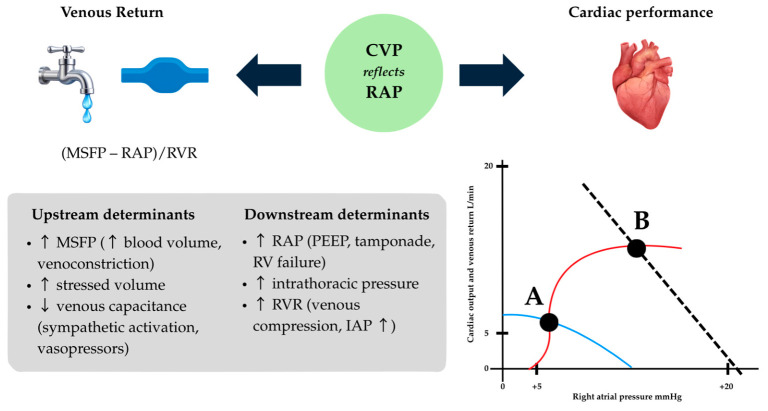
This schematic illustrates the interaction between venous return and cardiac performance according to the Guyton model of circulation. Venous return is determined by the gradient between mean systemic filling pressure (MSFP) and right atrial pressure (RAP), divided by resistance to venous return (RVR) = (MSFP − RAP)/RVR. Central venous pressure (CVP) is commonly used as an estimate of right atrial pressure (RAP) under appropriate physiological conditions, although the two are not fully interchangeable across all physiological and pathological conditions. Upstream determinants include factors increasing MSFP (e.g., blood volume, venoconstriction, stressed volume, reduced venous capacitance), while downstream determinants include factors increasing RAP, intrathoracic pressure, or RVR. The graph shows the venous return curve (blue) and the cardiac function curve (red); their intersection defines cardiac output. Point A represents baseline conditions, while a shift in the venous return curve (dashed line) leads to a higher equilibrium at point B. CVP reflects the interaction between venous return and cardiac function rather than intravascular volume alone, and increases in CVP may reduce the pressure gradient for venous return, potentially limiting cardiac output.

The historical development of CVP monitoring begins with early-20th-century experimental studies and foundational human research, which established CVP as a key parameter reflecting right atrial pressure and venous return [16,17]. The work of Frank and Starling demonstrated that ventricular stroke volume (SV) increases in response to increased end-diastolic filling, thereby defining the Frank–Starling mechanism and linking venous return and filling pressures to cardiac output (CO) [16]. Building on these concepts, Guyton formalized the relationship between CVP (approximating RAP), venous return, and CO within a unified circulatory model where CVP value emerges as the equilibrium between upstream drivers and downstream factors, with steady-state CO defined at the intersection of the cardiac function curve and venous return curve. Venous return, and thus CO, increases when mean systemic filling pressure (MSFP) rises, RAP falls, or resistance to venous return (RVR) decreases (e.g., through recruitment of collapsed capacitance vessels) [2,18]. Venous return is proportional to the pressure gradient between MSFP and RAP. Since CVP approximates RAP, an isolated increase in CVP reduces this gradient unless MSFP increases to a greater extent. Importantly, this relationship highlights that CVP is not a direct measure of circulating blood volume but rather a pressure that reflects the downstream conditions against which venous return must occur [4]. Therefore, increasing CVP alone does not improve venous return and may, in some cases, impair it. This concept provides a physiological explanation for why fluid administration aimed primarily at increasing CVP may fail to augment cardiac output and instead contribute to venous congestion (Figure 2).

The absolute value of CVP therefore reflects two main components: central venous blood volume and central venous compliance. An increase in intrathoracic venous blood volume, e.g., due to an acute fluid loading, or redistribution of blood from the peripheral to the central venous compartment, tends to raise CVP. Conversely, changes in venous compliance, driven largely by sympathetic tone and smooth muscle contraction in the venous wall, can increase CVP at constant volume by reducing the capacitance of the thoracic venous system. Physiologically, CVP is influenced also by multiple factors beyond intravascular volume, including intrathoracic pressure variations from respiration, right ventricular compliance, and valvular integrity. For this reason, CVP should be interpreted as a hemodynamic pressure signal reflecting the balance of these interacting factors rather than as a direct measure of central blood volume or cardiac preload (Figure 2) [1,2].

These physiological insights provided the theoretical foundation for the clinical adoption of CVP monitoring. The transition from experimental to clinical use accelerated in the 1950s, when CVP began to be routinely measured in human patients using internal jugular vein cannulation with water manometers [19]. Early volunteer studies documented the transient elevation and subsequent fall of CVP in response to fluid administration and hemorrhage, establishing the foundation for fluid resuscitation protocols that would dominate critical care for decades until the early 2000s [2,20].

In the years to come, a parallel body of research was quietly accumulating evidence that static CVP measurements used in isolation were poor predictors of FR [21,22]. In 2008, Marik et al. conducted a systematic review of 24 studies including more than 800 patients reporting only a very weak correlation between CVP and circulating blood volume and between baseline CVP and the change in stroke index or cardiac index after a fluid challenge [22]. Osman et al. reached similar conclusions in septic patients, showing that the Surviving Sepsis Campaign’s recommended CVP and PAOP targets failed to discriminate fluid responders from non-responders [23].

Consequently, from the early 2000s onward, dynamic indices derived from arterial waveform analysis, such as pulse pressure variation (PPV) and stroke volume variation (SVV), have gained increasing preference over static variables like CVP for predicting FR. These indices have been extensively validated and demonstrate good diagnostic accuracy under optimal conditions. However, as they are based on heart–lung interactions during mechanical ventilation, their reliability is highly dependent on specific physiological and technical prerequisites, including controlled mechanical ventilation, regular cardiac rhythm, and adequate tidal volumes. Their predictive performance is significantly reduced in the presence of low tidal volume ventilation, high PEEP, decreased lung or chest wall compliance, spontaneous breathing activity, or cardiac arrhythmias. Moreover, additional factors such as increased intra-abdominal pressure, peripheral vascular disease, and limitations in cardiac output monitoring may further impair their applicability at the bedside, ultimately restricting their use in a substantial proportion of critically ill patients [24,25,26,27].

From the early 2010s onward, the interpretation of CVP gradually shifted from its traditional role as an indicator of hypovolemia to a broader understanding of its value as a marker of venous congestion, especially in relation to renal dysfunction. This congestion reduces the trans-renal perfusion pressure gradient while increasing intra-tubular pressure, thereby impairing glomerular filtration despite preserved or even elevated systemic blood pressure. The implications were profound: pursuing CVP targets, often requiring aggressive fluid administration, could paradoxically exacerbate organ dysfunction by inducing venous congestion [28,29].

**Figure 3:**
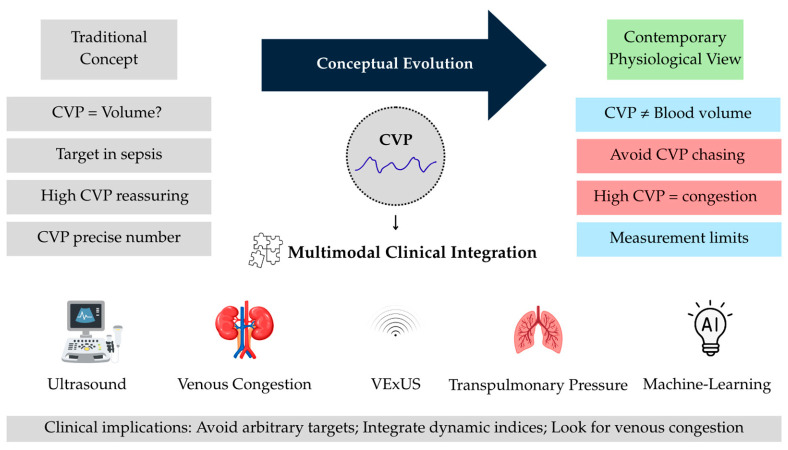
This schematic illustrates the transition from traditional interpretations of central venous pressure (CVP) to a modern, physiology-based framework and its integration into multimodal clinical assessment. Historically, CVP was considered a surrogate of intravascular volume, used as a resuscitation target in sepsis, and interpreted as a reassuring standalone numerical value. Contemporary understanding recognizes that CVP alone does not reliably reflect blood volume and should not be used as a target to “normalize.” Instead, elevated CVP is increasingly interpreted as a marker of venous congestion and right-sided loading conditions, while its clinical utility depends on contextual interpretation and awareness of measurement limitations. Within this modern framework, CVP is incorporated into a multimodal hemodynamic assessment that includes dynamic indices of fluid responsiveness, bedside ultrasound evaluation, venous congestion assessment (e.g., VExUS), and integration with respiratory mechanics such as transpulmonary pressure estimation. Emerging approaches, including machine-learning–based analysis of hemodynamic signals, may further enhance the interpretation of CVP within complex clinical scenarios.

In summary, the evolution of CVP from a physiologically grounded marker of RAP and venous return to a widely adopted clinical tool has been characterized by alternating phases of enthusiasm and skepticism. Initially embraced as a surrogate of intravascular volume and FR, CVP was later challenged both by experimental physiology and clinical evidence, which highlighted its limitations as a static predictor of preload and CO. More recently, its reinterpretation as an indicator of venous congestion and its association with renal and organ dysfunction has repositioned CVP from a stand-alone hemodynamic target to a contextual parameter within an integrated, multimodal approach to circulatory monitoring (Figure 3).

## 4. The Changing Role of CVP in Sepsis Resuscitation Guidelines

CVP historically played a central role in hemodynamic monitoring following the introduction of early goal-directed therapy (EGDT) for sepsis and septic shock in 2001, which was initially adopted as a standard resuscitation strategy. In this context, CVP was used to guide initial fluid administration, targeting 8–12 mmHg (12–15 mmHg in mechanically ventilated patients), alongside predefined goals for MAP and Central Venous Oxygen Saturation (ScvO_2_) [30]. However, over time, the concept of targeting fixed hemodynamic thresholds has been increasingly challenged. CVP remained part of resuscitation protocols for several years before being progressively de-emphasized in subsequent guideline updates [31]. From 2014 onward, the Rivers’ resuscitation protocol was increasingly challenged. Three large multicenter randomized trials—ARISE, ProCESS, and ProMISe—collectively enrolling thousands of septic patients, failed to demonstrate a mortality benefit of EGDT over usual care. These trials were conducted in a context of improved standard care, including earlier recognition of sepsis and timely antibiotic administration [32,33,34]. These findings indicated that rigid targets for CVP and ScvO_2_ did not improve survival in real-world practice, prompting guideline revisions.

Consequently, the 2016 Surviving Sepsis Campaign guidelines abandoned CVP and ScvO_2_ as mandatory resuscitation endpoints, favoring instead adequate initial fluid resuscitation followed by frequent reassessment of volume status and organ perfusion [35,36]. This transition marked a broader move away from protocolized, target-driven approaches toward a more flexible, physiology-based strategy. The 2021 update further emphasized that static variables such as CVP, heart rate, and arterial pressure are unreliable indicators of intravascular volume when considered in isolation and should not guide fluid therapy alone [15,37].

Recent updates from the Surviving Sepsis Campaign 2026 further reinforce and consolidate the multimodal and individualized approach, in which no single hemodynamic variable, including CVP, is sufficient to guide resuscitation, but may still contribute when interpreted within a broader clinical context [38].

In contemporary practice, fluid management in sepsis is increasingly based on dynamic and individualized assessment rather than predefined numerical thresholds. Repeated bedside evaluation of hemodynamic response and tissue perfusion has become central, integrating multiple parameters such as lactate kinetics, capillary refill time, echocardiographic findings, and markers of venous congestion. Within this framework, CVP is no longer pursued as a therapeutic target but may still provide complementary information when interpreted in a multimodal context [37].

## 5. Divergences from Evidence

### 5.1. Reasons of CVP Persistence in Clinical Practice

The FENICE study, an international observational study investigating fluid challenge practices in ICUs, found that static markers of preload—including CVP—were used in approximately one-third of fluid administration decisions [39]. Several factors may explain why CVP remains embedded in clinical practice despite being discouraged as a primary guide for fluid therapy. First, CVP is easily obtainable through a CVC that is often already in place for drug administration and blood sampling, making it a readily available and inexpensive signal compared with advanced monitoring tools. Second, many clinicians have been trained within paradigms that historically prioritized static preload surrogates (e.g., EGDT-based targets), and this entrenched mental model can persist even after guideline revisions. Third, although dynamic indices and functional hemodynamic tests are recommended for assessing FR, their applicability is often restricted by real-world conditions. Parameters such as PPV and SVV require controlled mechanical ventilation, regular cardiac rhythm, and adequate tidal volumes. In many critically ill patients, such as those with spontaneous breathing activity, arrhythmias, low tidal volume ventilation, or poor echocardiographic windows, these conditions are not met. As a result, the theoretical superiority of dynamic indices is frequently offset by limited feasibility at the bedside. In this context, clinicians may revert to simpler and universally available variables such as CVP, even when aware of their physiological limitations [40,41].

### 5.2. Physiological Interpretation

At a deeper level, the persistent use of CVP is also driven by its intuitive physiological interpretation as a simple bedside indicator of volume status and fluid tolerance. In everyday clinical practice, low values have traditionally been associated with hypovolemia, whereas high values have been interpreted as suggesting limited tolerance to further fluid administration. Although physiological evidence has shown that fluid responsiveness depends primarily on ventricular function and position on the Frank–Starling curve rather than on static pressure values, this simplified conceptual approach remains attractive in time-pressured clinical settings [16]. Moreover, CVP provides information that extends beyond FR alone. When interpreted dynamically and integrated with other variables, it may contribute to the assessment of right ventricular function, venous congestion, and temporal trends. For this reason, many clinicians continue to incorporate CVP within a multimodal hemodynamic evaluation rather than abandoning it entirely [42]. Ultimately, the persistence of CVP reflects not a lack of evidence, but the complexity of translating physiological knowledge into feasible bedside practice.

## 6. Modern CVP Uses

### 6.1. Venous Congestion and Organ Perfusion

Modern interest in CVP concerns venous congestion and organ perfusion rather than prediction of FR. Elevated CVP is consistently associated with acute kidney injury, impaired organ perfusion, and worse outcomes, and there is strong physiological plausibility that venous congestion contributes causally in at least a subset of patients [43].

From a hemodynamic perspective, the clinical relevance of CVP lies not only in its role as a surrogate of RAP but also in its contribution to organ perfusion pressure. Organ perfusion is determined by the gradient between upstream arterial pressure and downstream venous pressure, commonly approximated by mean perfusion pressure (MPP = MAP − CVP). However, this simplified relationship should not be interpreted as a universal organ-perfusion equation, because organ-specific factors may substantially modify effective perfusion, particularly in the kidney. In renal circulation, the effective perfusion gradient is further influenced by intra-abdominal pressure, renal venous pressure, autoregulatory mechanisms, and microcirculatory injury. Accordingly, CVP should be considered one component of a broader congestion-perfusion framework. Even so, elevated CVP may still reduce the effective pressure gradient for organ perfusion and contribute to impaired organ function despite apparently preserved MAP [43,44,45,46,47]. High CVP, in fact, reflects backward failure of the right heart and systemic venous congestion transmitting backward pressure to the renal veins and microcirculation, where it alters the pressure gradients that normally drive glomerular filtration and promotes kidney injury even in the presence of apparently satisfactory mean arterial pressure [29,43,48]. Multiple observational studies, including large international sepsis cohorts, have shown that a more positive cumulative fluid balance during the first days of critical illness is associated with increased mortality and organ dysfunction in septic patients [49,50,51].

In this context, CVP reflects the downstream pressure component of the circulation and may provide clinically relevant information on venous congestion and organ perfusion when integrated with other hemodynamic and clinical variables.

### 6.2. VExUS

Ultrasound-based approaches such as the venous excess ultrasound (VExUS) grading system integrate inferior vena cava diameter with Doppler analysis of hepatic, portal, and intrarenal venous flow patterns to assess the transmission of right atrial pressure to abdominal organs. In this context, CVP provides a global estimate of RAP, whereas Doppler-derived venous waveforms reflect the hemodynamic consequences of venous hypertension on organ-specific venous circulation. Rather than being interchangeable, these techniques should be considered complementary: CVP represents an upstream hemodynamic variable, while VExUS characterizes the downstream expression of systemic venous congestion. Their integration may enable the construction of integrated “congestion profiles,” potentially improving the identification of patients at risk of venous hypertension–related organ dysfunction [52,53].

This interpretation is consistent with recent ESICM hemodynamic monitoring guidance, which supports the use of bedside ultrasound and venous congestion assessment within a multimodal evaluation strategy for fluid management decisions [54]. In fact, contemporary hemodynamic assessment increasingly distinguishes between “fluid responsiveness” and “fluid tolerance,” recognizing that elevated venous pressures may contribute to organ dysfunction even in the absence of overt intravascular volume overload [55,56]. In this setting, VExUS may provide complementary information regarding the organ-level consequences of venous congestion. Recent studies have demonstrated that VExUS correlates closely with invasively measured RAP and may outperform inferior vena cava diameter or collapsibility alone in identifying elevated right-sided filling pressures [53,57]. However, VExUS represents an intermittent and not continuous assessment of congestion severity and venous outflow impairment. Importantly, discordance between CVP and VExUS findings may itself carry clinical significance. Elevated CVP with preserved venous Doppler patterns may reflect early hemodynamic congestion without established organ involvement, whereas abnormal VExUS findings despite modest CVP values may suggest impaired right ventricular–pulmonary arterial coupling or clinically relevant venous congestion [58,59]. Taken together, CVP and VExUS provide complementary information: the former reflects central venous pressure and waveform dynamics, whereas the latter describes the peripheral vascular consequences of venous hypertension and may better capture clinically relevant fluid intolerance.

### 6.3. Waveform Morphology and Right Heart Pathophysiology

In contemporary critical care, CVP is often reduced to a single numerical value, typically the mean pressure, which is then interpreted as a surrogate of intravascular volume or preload. However, this simplification overlooks a key aspect of CVP physiology: it is inherently a dynamic signal reflecting time-varying pressure changes within the right atrium and central venous system. This oversimplified interpretation of CVP as merely a static numerical value likely contributes to many of the misconceptions surrounding its clinical utility and to its perception as a potentially misleading parameter. In contrast, the analysis of CVP waveform morphology provides direct insight into right-heart function and atrial-ventricular interactions, as each component of the waveform corresponds to specific cardiac events. From this perspective, CVP waveform analysis represents one of the few applications in which CVP retains a direct and physiologically grounded interpretation, rather than acting as an indirect or surrogate marker (Table 1) [2]. Importantly, these waveform features are not merely adjunctive observations but represent real-time expressions of underlying cardiac physiology. When integrated with echocardiographic findings and the clinical context, waveform analysis may contribute to the identification of right-sided cardiac abnormalities and improve bedside hemodynamic interpretation.

In this context, CVP should not be viewed as misleading per se, but rather as a physiological signal whose clinical value depends on how it is interpreted. While its mean value alone may be insufficient and potentially misleading, the waveform itself provides meaningful information that cannot be captured by a single numerical parameter. This distinction reinforces the concept that the limitations of CVP arise not from the variable itself, but from its oversimplified interpretation [14].

### 6.4. Role During Fluid Challenge

In contemporary practice, CVP could be used as complementary information to the interpretation of the hemodynamic response to fluid administration [60]. Serial assessment of CVP may help clarify how the patient is tolerating a fluid challenge, particularly when interpreted in relation to changes in cardiac output, arterial pressure, tissue perfusion, and signs of venous congestion. In this context, an increase in CVP accompanied by improved cardiac output may reflect effective preload recruitment. In contrast, a disproportionate rise in CVP without hemodynamic benefit may indicate limited cardiac tolerance, impaired right ventricular function, or an early shift toward venous congestion [61]. This distinction is clinically relevant because fluid responsiveness alone does not establish that further fluid administration is appropriate. Recent evidence highlights the importance of integrating preload responsiveness with fluid tolerance, recognizing that some patients may transiently increase cardiac output while simultaneously developing harmful venous congestion [62]. Within this framework, CVP trends should not be interpreted as predictors of response, but as part of the bedside evaluation of the balance between potential benefit and potential harm during fluid therapy. Accordingly, CVP is best regarded as a complementary monitoring variable during fluid challenge: not a decision-making endpoint in itself, but a physiological signal that may help define the limits of fluid administration and support a more cautious, individualized resuscitation strategy, as it may also serve as a marker of the cost of fluid administration rather than its potential benefit.

### 6.5. Emerging and Expanding Applications

In recent years, an emerging and still investigational application of CVP has been proposed, extending its role beyond conventional hemodynamic monitoring. Specifically, respiratory variations in the CVP waveform have been explored as a surrogate for estimating changes in pleural pressure and, consequently, transpulmonary pressure (TPP), with the aim of assessing the mechanical stress imposed by positive pressure ventilation—particularly in patients with acute respiratory distress syndrome (ARDS), where limiting ventilator-induced lung injury (VILI) is a key objective [63,64,65].

TPP, defined as the difference between alveolar pressure and pleural pressure, represents the true distending pressure of the lung. In clinical practice, pleural pressure is typically estimated using esophageal pressure obtained via an esophageal balloon catheter. However, this technique is invasive, technically demanding, and not widely adopted outside specialized centers due to challenges related to positioning, calibration, and interpretation [66,67,68]. In this context, CVP has been proposed as a more readily available surrogate signal. Because the central veins and right atrium are located within the thoracic cavity, respiratory swings in the CVP waveform (ΔCVP) may partially reflect changes in pleural pressure, provided that cardiac oscillations are appropriately filtered and the venous compartment remains mechanically coupled to intrathoracic pressure [12,13]. To improve signal interpretation, several signal-processing approaches combining CVP waveform analysis with ECG-based filtering techniques have been proposed to isolate respiratory-related components from cardiac pulsatility [12,69,70,71]. Nevertheless, CVP-derived signals cannot reliably estimate absolute pleural pressure and should not be considered interchangeable with esophageal pressure measurements. Current physiological and clinical evidence suggests that ΔCVP may occasionally approximate respiratory pleural pressure changes, but with substantial variability related to patient position, thoracic compliance, venous mechanics, and signal quality [72,73,74,75,76]. Direct comparative and experimental studies suggest that ΔCVP shows weaker and more variable agreement with esophageal pressure–derived estimates of pleural pressure than other invasive surrogates, and often underestimates pleural pressure swings, particularly in the presence of spontaneous breathing or altered thoracoabdominal mechanics [12,69,72]. Accordingly, recent clinical guidance continues to support esophageal pressure monitoring as the reference method when pleural pressure estimation is required to guide ventilatory management and transpulmonary pressure–based strategies [77]. Although experimental and preliminary clinical studies suggest potential feasibility in selected settings, CVP-derived pleural pressure estimation remains investigational, has not been validated for routine clinical decision-making, and requires further physiological and clinical validation before integration into standard ventilatory management strategies.

Emerging technologies may further enhance the clinical interpretation of CVP in the future. In particular, artificial intelligence and machine-learning approaches are being explored to integrate continuous hemodynamic signals, ultrasound data, and clinical variables to identify patterns of venous congestion and predict fluid responsiveness or organ dysfunction. Such multimodal analytical frameworks could allow CVP to be incorporated into dynamic decision-support systems, improving the precision of hemodynamic management in critically ill patients. However, these applications remain exploratory, and their routine use in critical care has not yet been established [78].

Finally, CVP retains clinical value when interpreted within a multimodal hemodynamic assessment. In situations where dynamic indices are technically unavailable or unreliable, CVP may still serve as one contextual variable alongside bedside echocardiography, lactate trends, urine output, and other markers of tissue perfusion [62]. Rather than representing a standalone decision-making tool, CVP should therefore be considered part of a hierarchical and integrated approach to hemodynamic monitoring in the critically ill patient.

## 7. Conclusions

CVP should not be interpreted as an isolated target for normalization, but as a contextual hemodynamic signal whose meaning depends on waveform morphology, respiratory conditions, right-heart function, organ perfusion, and the broader clinical scenario. CVP may still provide clinically meaningful information when interpreted within an integrated physiological and multimodal hemodynamic framework.

## Figures and Tables

**Table 1:** This table summarizes characteristic CVP waveform abnormalities associated with specific cardiac and hemodynamic conditions. In atrial fibrillation, the absence of organized atrial contraction results in loss of the a-wave. Cardiac tamponade is characterized by prominent x-descent and blunted or altered y-descent due to impaired ventricular filling. Junctional rhythms may produce cannon a-waves from atrioventricular dissociation. Pericardial constriction typically shows a rapid y-descent reflecting abrupt early diastolic filling. Tricuspid regurgitation is associated with prominent v-waves due to systolic backflow into the right atrium. In right ventricular failure or pulmonary hypertension, elevated CVP values are often accompanied by accentuated a-waves and attenuated y-descent, reflecting impaired right ventricular compliance and filling.

CVP Waveform and Clinical Meaning	
**Atrial Fibrillation**	**Cardiac Tamponade**
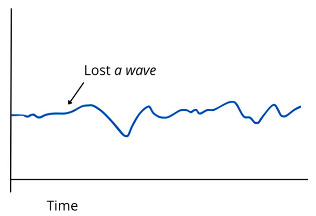	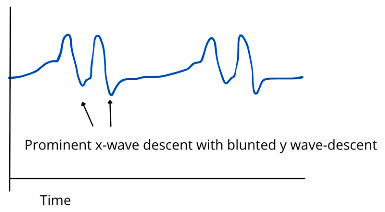
Absence of *a*-waves	Prominent *x*-descent with blunted *y*-descent
**Junctional Rhythm**	**Pericardial Constriction**
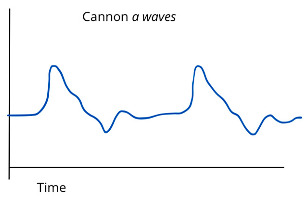	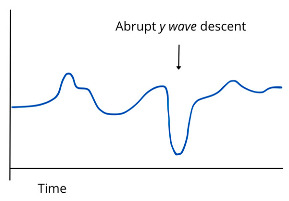
Cannon a-waves	Steep abrupt *y*-wave descent
**Tricuspid Regurgitation**	**Right ventricular failure/pulmonary hypertension**
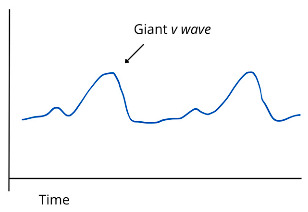	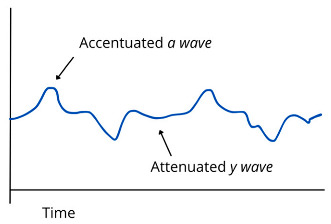
Giant *v*-waves	Elevated mean CVP values

**Table 2:** This table summarizes respiratory, cardiac, vascular, hemodynamic, technical, and interpretative factors that influence central venous pressure (CVP), potentially altering its relationship with true intravascular volume and right atrial pressure, along with their underlying mechanisms and clinical implications.

Category	Specific Cause	Mechanism of Inaccuracy	Clinical Implication
**Respiratory Factors**	High PEEP/mechanical ventilation	Increase intrathoracic pressure → artificially elevates CVP	Overstimation of preload and volume status
	Spontaneous breathing efforts	Negative intrathoracic pressure swings → decreases CVP	Underestimation of filling pressures
	Dynamic hyperinflation/auto-PEEP	Sustained elevation of intrathoracic pressure	Persistent CVP overestimation
	Poor timing of measurement (not at end-expiration)	Respiratory variations distort true value	Misleading single-point measurements
**Cardiac** **Factors**	Tricuspid regurgitation	Systolic backflow → large v-waves and elevated mean CVP	Misleading interpretation of preload or transmural filling conditions
	Right ventricular dysfunction	Elevated RV filling pressures	High CVP despite low preload
	Cardiac tamponade	Impaired transmural ventricular filling despite elevated intracardiac pressures	High CVP despite low preload
	Constrictive pericarditis	Impaired diastolic filling	Elevated CVP with abnormal waveform
	Atrial fibrillation	Loss of a-wave	Difficult waveform interpretation
	Atrioventricular dissociation	Cannon a-waves	Intermittent CVP spikes
**Vascular Factors**	Reduced venous compliance (↑ sympathetic tone)	Same volume → higher pressure	CVP overestimates volume
	Increased intra-abdominal pressure	Impairs venous return → elevates CVP	False impression of volume overload
	Venous obstruction (e.g., SVC syndrome, thrombosis)	Impaired drainage → elevated upstream pressure	CVP not reflecting right atrial pressure
**Volume Status & Hemodynamics**	Hypervolemia	True increase in venous pressure	May reflect congestion rather than preload reserve
	Hypovolemia with high intrathoracic pressure	Opposing effects distort CVP	Unreliable assessment of volume status
	Redistribution of blood (venoconstriction)	Centralization of volume	Elevated CVP without true volume increase
**Technical Factors**	Incorrect transducer leveling	Reference point error	Systematic over- or underestimation
	Failure to zero to atmospheric pressure	Calibration error	Inaccurate absolute values
	Catheter malposition	Non-central measurement	Invalid CVP reading
	Air bubbles/clot in line	Signal damping or artifact	Distorted waveform and values
	External compression of catheter	Artificial pressure elevation	False high CVP
**Interpretation Errors**	Use of single absolute value	Ignores dynamic and contextual factors	Misleading clinical decisions
	Ignoring waveform morphology	Loss of diagnostic information	Missed cardiac pathology
	Using CVP to predict fluid responsiveness	Poor correlation with preload reserve	Inappropriate fluid administration

## Data Availability

No new data were created or analyzed in this study.

## Abbreviations

The following abbreviations are used in this manuscript:

AF	Atrial Fibrillation
ARDS	Acute Respiratory Distress Syndrome
CO	Cardiac Output
CP	Constrictive Pericarditis
CVC	Central Venous Catheter
CVP	Central Venous Pressure
EGDT	Early Goal-Directed Therapy
FR	Fluid Responsiveness
MAP	Mean Arterial Pressure
MPP	Mean Perfusion Pressure
MSFP	Mean Systemic Filling Pressure
PAOP	Pulmonary Artery Occlusion Pressure
PEEP	Positive End-Expiratory Pressure
PPV	Pulse Pressure Variation
PT	Pericardial Tamponade
RAP	Right Atrial Pressure
RV	Right Ventricle
RVR	Resistance to Venous Return
ScvO_2_	Central Venous Oxygen Saturation
SVC	Superior Vena Cava
SV	Stroke Volume
SVV	Stroke Volume Variation
TPP	Transpulmonary Pressure
VExUS	Venous Excess Ultrasound Score

## Appendix A

**Table A1:** Supplementary.

SANRA Item	Description	Addressed in Manuscript
1. Justification of the article’s importance	The review discusses the evolving interpretation of CVP in modern critical care and the gap between historical practice and current physiological evidence.	Introduction
2. Statement of concrete aims or formulation of questions	To reassess the physiology, limitations, and contemporary clinical role of CVP, focusing on fluid responsiveness, venous congestion, and multimodal monitoring.	Introduction
3. Description of the literature search	Databases, keywords, and overall search strategy are described	Introduction
4. Referencing	Relevant and updated literature, including major studies and guidelines, is appropriately cited.	Throughout manuscript
5. Scientific reasoning	Relevant and updated literature, including major studies and guidelines, is appropriately cited.	Throughout manuscript
6. Appropriate presentation of data	Tables, figures, and structured sections improve clarity and readability.	Tables, Figures, Sections
